# Odontological Society of Pennsylvania

**Published:** 1884-02

**Authors:** 


ARTICLE IV.

### ODONTOLOGICAL SOCIETY OF PENNSYLVANIA.

The regular meeting of the Odontological Society of Pennsylvania was held at the office of Dr. E. C. Kirk, 1602 Arch Street, Saturday evening, November 3rd, 1882. President Truman in the chair.

Dr. Kirk read the following paper upon the

### RELATION OF FOOD TO THE TEETH.

So much of our time is devoted to the repair of carious teeth by the various mechanical methods of filling, that when we are fortunate or skillful enough to succeed in arresting decay by these means, we are too apt to feel that we have done all our patients have required of us, but should our attention cease at this point?

It is my purpose this evening to direct your thought to some points relating to the proper nutrition of the teeth, a disregard for which, in my belief, is one of the most fruitful predisposing causes of caries. For the past eighteen months I have had under my care the mouths of between three and four hundred children, pupils of the Pennsylvania Institution for the Deaf and Dumb, and to the statistics furnished me by the superintendent relating to food, clothing, hygienic conditions, &c., together with my personal examination and supervision of their mouths and teeth, I am largely indebted for what knowledge I have obtained relative to the positive effects of proper food on the teeth. It is only where a considerable number of individuals are provided with the same quality and amount of food, and all the other conditions of living are as nearly as possible equal, that we can hope to obtain reliable data from which to study such a subject successfully and intelligently, The teeth of these deaf mute children present many points of interest, and a close observation of them has served to fix in my mind the conviction that if we should successfully combat the ravages of dental caries our attentions must not cease after we have restored by filling the tissue which has been destroyed, but by instructing our patient, and especially the parent or guardian, in the case of children, as to the proper course of diet and mode of living to be pursued during the period of calcification of the bones and teeth, we can secure for them the kind of nourishment required and insure practically sound dental organs in many cases.

On examining the mouths of a class of children, recently admitted to the institution, the teeth present no peculiarities worthy of special notice, except in most cases a lack of cleanliness and an average amount of caries, not greater than we would expect to find in the mouths of the same number of children where no previous dental care had been given, and where brush and powder were unknown; the tooth structure is often quite soft and the decay is largely of the white variety, such as occurs in teeth deficient in mineral constituents. They come to the institution from the lower walks of life as a rule, their parents in many cases being poor and unable to provide them with proper food and care; as a natural result there are many whose dentures are riddled with caries, pulps exposed, and the teeth apparently melting away. In those whose deafness is acquired through disease, viz: scarlatina, measles, cerebro-spinal meningitis, the teeth frequently bear evidences of the high febrile conditions through which the patient has passed, by pits in the enamel, crimped edges, defective form, imperfect development or soft structure. The majority of the pupils are admitted between the ages of ten and twelve years. After a year's residence in the institution, during which time they are given excellent care in all that relates to their physical welfare, a marked improvement will be observed in the character of their teeth, they will be found exceedingly hard and dense, making the wear and tear on cutting instruments very great; excavators and chisels have to be tempered to the point of brittleness to be effective in preparing cavities, otherwise they will not cut the extremely hard structure. They are firmly set in their sockets, making them unusually difficult to extract. These are not visionary observations, but the result of repeated tests and examinations carefully made. But the most conclusive evidence which I have met with of the value of their diet table, so far as the nutrition of their teeth is concerned, is the unusual number of cases of arrested caries and the formation of the so-called secondary dentine.

You are well aware that such action can only take place under the most favorable conditions. Nature never makes an attempt to repair a carious tooth unless she is compelled to by an excess of tooth and bone forming pabulum in the blood. I have seen from time to time cases in private practice where this action had taken place, but they occur rarely. Among the pupils of the Deaf and Dumb Institution it is quite common, not only in the permanent dentures but also in the temporary sets. As showing still further the abundance of bone-forming material with which their blood is supplied, I have removed in two cases large pulp nodules from the sixth year molars of children not over eleven years of age; these I considered unique, as I had never met with this formation before in patients so young.

Oatmeal and wheat grits have been recommended, and justly so as a proper food for hardening the teeth. on account of the abundance of phosphatic material which exists in the outer covering of the grain; still, damage can be done in the improper use of these. I was afforded an opportunity by Dr. Eisenbrey to examine the mouth of a patient of his, a boy perhaps fifteen years of age, whose teeth looked as though he had lived principally on candy; they were soft and sensitive with an eroded appearance of the enamel, which, though perfectly white and clean, was rough and softened as though it had been dipped in a strong acid. The young man was excessively fat. Upon making inquiry of his father, who was present, as to his son's usual diet, at the same time calling his attention to the eroded condition of his teeth, he said he was sure his food could have nothing to do with it, for he was very fond of oatmeal, and that was always highly recommended as beneficial to the teeth." Upon asking him if he used much sugar with it, he said : "Oh yes! in large quantities—he fairly covers it up in sugar." Here, then, was the secret: the mass of oatmeal was sweetened to an extent almost nauseating, and its nutritive qualities were more than balanced by the fat and heat producing properties of the sugar. His pampered and petted existence, together with a lack of sufficient exercise, had brought about a condition of obesity amounting to disease, and wholly incompatiable with a healthy denture. Nutrition comprehends digestion, absorption, respiration, circulation and assimilation ; upon the harmonious action of these separate functions depend life, growth and health. While the object of my paper has been simply to show the effect that a fixed and uniform diet has had upon the teeth of a given number of children, I would not ignore the importance of proper hygienic measures and methods of living, without an observance of which, proper digestion, absorption, and assimilation are impossible. Nor would I be understood as asserting that the diet list which I have quoted is the only proper one to follow. I have brought it to your notice because I believe it to be an important but by no means the only factor in producing good, strong and dense teeth at the Deaf and Dumb Institution. It does present some features which, taken in connection with the results achieved, form a basis for some important deductions. The food is plain, well-cooked and wholesome, there is a a total absence of sweetmeats and pastry, the amount of animal food bears but a small proportion to the vegetable, a liberal amount of whole grain, *i. e.*, that which has not had the outer or phosphatic envelope removed, is supplied in the shape of oat and wheat grits, there is a total absence of fried and greasy food, and last a liberal supply of milk. All this with a good appetite and thorough digestion and assimilation goes far toward accounting for the unmistakable improvement which certainly takes place in the quality of their teeth. Much could be said and ought to be said upon the evils of badly-ventilated school-rooms and sleeping apartments, late hours and constant overstimulation of the brain by an endless variety of exciting causes, or, what is equally bad, the high pressure system of education so much in vogue at the present time, by which children are loaded down with an amount of mental work which the majority of them are physically incapable of carrying; this too, at a time when their most rapid growth is taking place, diverting from its proper function the nerve force which should have been applied to physical development. The results of this state of affairs we see around us daily, a finely-developed intellect, a highly-cultivated and educated brain united to a body, the principal object of which seemed to be to make life a burden.

The data which I have presented to you, and the observations upon them have occupied my thought and interested me for some time. I bring them to you hoping they may throw some light upon and in some degree add to our knowledge of the causes of dental caries, and especially its preventive treatment.—*Dr. Kirk, in Transactions of Odontological Society of Pennsylvania, Dental Office and Laboratory.*

